# Phenotyping moderate asthma earlier by analyzing induced sputum eosinophils, blood eosinophils, and atopy: a cross-sectional comparison with severe asthma

**DOI:** 10.36416/1806-3756/e20240354

**Published:** 2026-03-05

**Authors:** Marcia Margaret Menezes Pizzichini, Cristiane Cinara Rocha, Emilio Pizzichini

**Affiliations:** 1. Programa de Pós-Graduação em Ciências Médicas, Universidade Federal de Santa Catarina - UFSC - Florianópolis (SC) Brasil.; 2. Núcleo de Pesquisa em Asma e Inflamação das Vias Aéreas - NUPAIVA - Universidade Federal de Santa Catarina - UFSC - Florianópolis (SC) Brasil.

**Keywords:** Asthma, Sputum, induced, Biomarkers, Quality of life, Biologic Medicines

## Abstract

**Objective:**

There has been limited research comparing the inflammatory characteristics of moderate and severe asthma in Brazil. To describe and compare the phenotypes of moderate asthma (MA) and severe asthma (SA) in Brazilian patients.

**Methods:**

In this cross-sectional study, we defined SA per the 2014 European Respiratory Society/American Thoracic Society criteria and MA per the 2018 GINA criteria. No participants had other lung diseases, recent respiratory infection, or a recent exacerbation. Asthma was characterized and phenotyped by using validated tests.

**Results:**

Compared to MA (n = 55), SA (n = 51) had more hospital (p < 0.001) and ICU admissions (p = 0.01), worse fixed airflow limitation (FEV_1_/FVC post-BD = 0.61; p < 0.001), poorer asthma control (p = 0.001), and reduced quality of life. However, this was not associated with higher activity of inflammation as measured by IS and Beos counts, which were similar. In addition, of the 19 patients with MA who reported ≥ 1 exacerbation in the last year, 12 (63.1%) had residual eosinophilia in induced sputum, compared with 22 (62.8%) of the 35 such patients with SA. The correlation between IS eosinophils and Beos was strong (rs = 0.75, R^2^ = 0.35; p < 0.001), though agreement was moderate. T2 phenotypes were predominant (76% in both groups). However, in the MA group, the prevalence of eosinophilic allergic asthma was higher than that of nonallergic asthma (56% vs. 20%; p < 0.001). In the SA group, those phenotypes had a similar prevalence (41% and 35%, respectively).

**Conclusions:**

Our analysis indicates that most subjects with SA or MA have the T2-high asthma phenotype, primarily allergic eosinophilic, in MA. Significant residual eosinophilic airway inflammation and recurrent exacerbations in some patients with MA suggest the need for further research on the optimal timing for

## INTRODUCTION

Asthma is a complex, heterogeneous disease.^1^ Its complexity arises from the interplay of genetic factors, environmental exposure, and various irritants affecting the airways, including allergens, viruses, bacteria, and tobacco smoke.^2^ Asthma can begin during fetal development and can continue throughout life, with symptoms potentially emerging at various ages, including older adulthood.^3^ Asthma is also deemed heterogeneous because of the involvement of multiple cell types and their proinflammatory products,^4^ ^,^ ^5^ leading to differences in clinical manifestations among patients. Most cases of asthma begin in childhood, tend to be mild, and are managed with low doses of inhaled corticosteroids (ICS) in combination with long-acting bronchodilators (LABAs).^6^ Only 3-5% of individuals with asthma experience severe asthma (SA), which may not respond to higher doses of ICS and LABAs or oral corticosteroids (OCS), resulting in frequent exacerbations, and hospitalizations, as well as, in many instances, irreversible airway damage and remodeling.^7^ ^,^ ^8^

In the past three decades, significant advances have been made in our understanding of SA, leading to improved management strategies and the development of biologic treatments that can, in many cases, achieve complete control and even remission of the disease in approximately a third of patients.^9^ Although the pathophysiology and endotypes of SA are not yet fully understood, it has been categorized into two main phenotypes. These phenotypes, classified as the T2-high phenotype (comprising allergic eosinophilic and nonallergic eosinophilic asthma) and the T2-low phenotype (comprising noneosinophilic and paucigranulocytic asthma), assist in selecting the most appropriate treatment for each patient.^10^ The neutrophilic phenotype, observed through induced sputum (IS) examination and often categorized by some researchers as another T2-low phenotype, requires further clarification. There is ongoing debate regarding its classification-whether it should be considered a distinct phenotype or simply a secondary condition associated with an alternative specific phenotype. A better understanding of its mechanisms and implications is essential for advancing research in this area.^11^ Conversely, several contemporary asthma guidelines^8^ ^,^ ^12^ categorize asthma into steps that reflect an increasing severity, indicating the escalating treatment necessary to achieve control. Although this framework is informative, it can be misleading, because asthma severity often exists on a continuum rather than being defined by distinct levels. In addition, longitudinal variability, a key characteristic of asthma, is marked by periods of worsening and improvement that require adjustments in treatment. Therefore, distinguishing between moderate MA and SA is not always easy. 

Although numerous randomized controlled trials on biologics for SA have included patients with MA and SA, none have specifically examined the differences in phenotypes between those two categories.^13^ ^-^ ^16^ However, the results of those trials suggest that some patients with MA may also require biologic treatment due to persistent residual airway inflammation. Timely intervention in these cases could enhance remission rates,^17^ an increase in which is associated with lower baseline severity, shorter disease duration, and fewer comorbidities. However, this should not be construed as justification for prescribing biologics unnecessarily; further studies are needed in order to confirm this hypothesis.

Despite the several international publications on the characteristics of SA, research on this topic in Brazil remains limited.^18^ ^-^ ^21^ Therefore, in this descriptive cross-sectional study, nested within a cohort study, we aimed to compare the phenotypes of MA and SA, given that they represent adjacent steps in asthma severity and treatment. Hence, we assessed the inflammatory characteristics of both groups by measuring IS cellularity, blood eosinophil (bEOS) levels, and atopy, while classifying participants by asthma phenotype.

## METHODS

### Design and patients

This was a cross-sectional, nonprobabilistic study. The primary endpoint was to describe the distribution of various asthma phenotypes in MA and SA. Secondary outcomes included the correlation between IS eosinophils and blood eosinophilia. Patients from 18 to 70 years of age with MA or SA, matched by age and sex, were consecutively recruited from the SA outpatient clinics of the University Hospital operated by the *Universidade Federal de Santa Catarina* (UFSC, Federal University of Santa Catarina), in the city of Florianópolis, Brazil, and from other specialized clinics in the city. Those patients were referred to the UFSC Center for Research on Asthma and Airway Inflammation. All of the participants had experienced asthma symptoms for more than one year. The diagnosis of asthma was confirmed objectively by demonstrating variable airflow limitation.^22^ We defined SA according to the international criteria established by the European Respiratory Society/American Thoracic Society^23^ and in Brazilian national guidelines,^8^ whereas we defined MA on the basis of the 2018 GINA criteria for step IV treatment.^24^ Therefore, SA was defined as that confirmed by an objective method, with good adherence to treatment and inhalation technique, and which, despite the elimination or minimization of factors associated with a lack of disease control, required high doses of ICS (budesonide ≥ 1,600 µg or equivalent), together with a second controller drug (a LABA, a long-acting muscarinic antagonist, or a leukotriene receptor antagonist) or OCS, on ≥ 50% of the days in the previous year, to maintain control of the disease, or as that which remains uncontrolled, due to the intrinsic severity of the asthma.^23^ In contrast, MA was defined as that requiring medium-dose ICS+LABA and a short-acting β_2_ agonist for rescue or medium-dose ICS+formoterol for maintenance and low-dose ICS+formoterol for rescue.^24^ The diagnosis of SA was made not only at enrollment in the study; all patients had been diagnosed with the condition at least 12 months before the study onset.

All of the patients evaluated had received a diagnosis of asthma more than a year before enrollment in the study, were either never smokers or former smokers with a smoking history of ≤ 10 pack-years who had quit smoking at least 12 months prior, and had had their inhaler technique confirmed as being appropriate. None of the patients had other lung diseases, respiratory infections, or recent exacerbations (within the past two months), or a history of noncompliance with treatment. They were screened to identify, reduce, or eliminate exposures that could negatively impact asthma control, including environmental and occupational exposures, as well as allergens and medications. In addition, all participants had their comorbidities identified, treated, or minimized, including chronic rhinosinusitis (with or without nasal polyps), vocal cord dysfunction, dysfunctional breathing, gastroesophageal reflux, bronchiectasis, obesity, obstructive sleep apnea, and anxiety/depression, given that those conditions are known to worsen asthma control. The study was approved by the local research ethics committee (Reference no. CAAE: 55534616.4.0000.0121), and all participating patients gave written informed consent.

### Definitions

Asthma phenotypes were divided into T2-high (allergic eosinophilic or nonallergic eosinophilic) and T2-low (noneosinophilic or paucigranulocytic).^8^ ^,^ ^24^ Sputum induction was performed on more than one occasion prior to the study visit (in the cohort study) for all patients with SA and for most of those with MA, particularly for those without sputum eosinophilia. Asthma was categorized as allergic eosinophilic if the participant had a positive skin prick test, together with ≥ 2.0% IS eosinophils or ≥ 150 bEOS/mm^3^. Nonallergic eosinophilic asthma was defined by a negative skin prick test accompanied by ≥ 2.0% IS eosinophils or ≥ 150 bEOS/mm^3^. Noneosinophilic or paucigranulocytic asthma was characterized by normal total cell counts and normal eosinophil/neutrophil counts in IS.^8^ Patients with ≥ 70% neutrophils and elevated total cell counts in IS were not classified as having a neutrophilic phenotype, given that there is ongoing debate about whether this represents a distinct phenotype or a misconception.^11^

### Procedures

Patient characteristics were documented by a structured questionnaire. Allergy skin tests were performed by the modified skin prick technique^25^ with 14 common allergen extracts. Asthma control was assessed with the six-item Asthma Control Questionnaire (ACQ-6), developed by Juniper et al.^26^ Asthma quality of life was assessed using the self-administered Standardized Asthma Quality of Life Questionnaire, or AQLQ(S).^27^ ^,^ ^28^ These questionnaires have been translated to Portuguese and validated for use in Brazil. Spirometry was performed, according to the American Thoracic Society standards,^28^ before and 15 min after inhalation of 200 µg of salbutamol through a metered-dose inhaler (Aerochamber; Trudell Medical International, London, ON, Canada). Reference values were taken from Crapo et al.^29^ Methacholine challenge tests were carried out in patients with normal or near-normal spirometry results, by the tidal breathing method.^30^ Sputum was induced and processed for total and differential cell counts by the methods described by Pizzichini et al.^31^ The presence of comorbidities was assessed by digestive endoscopy and, when required, by pHmetry, high-resolution chest computed tomography, examination by an otolaryngologist, plethysmography, and polysomnography, as well as evaluation by an endocrinologist and a psychiatrist. To obtain blood counts, blood samples were collected from patients by standard procedures.

### Statistical analysis

Descriptive statistics were used, including means with 95% confidence intervals, medians with interquartile ranges, and proportions, when appropriate. Differences between groups were examined by independent t-tests or nonparametric tests for two independent samples, as appropriate. Associations between the various markers were quantified by logistic regression analysis. The level of agreement between IS eosinophilia and blood eosinophilia was determined by calculating the kappa statistic. The significance level accepted was 95%, and all tests were two-tailed. Data were analyzed with the IBM SPSS Statistics software package, version 29.0 for Windows (IBM Corporation, Armonk, NY, USA).

## RESULTS

### Demographic and clinical characteristics of the study population

A total of 106 patients with asthma were recruited for the study. Of those, 51 (48.1%) were classified as having SA. Eleven patients (10.4%) were unable to produce adequate sputum for examination-6 from the MA group and 5 from the SA group. The main demographic and clinical characteristics of the subjects are detailed in Table 1. 

**Table 1:** Demographic and clinical characteristics of subjects.

Characteristic	Asthma intensity*		p
	Moderate	Severe	
	(n = 55)	(n = 51)	
Age (years), mean (95% CI)	51.6 (47.9-55.2)	50.2 (46.9-55.0)	0.8
Male, n (%)	8 (14.5)	12 (23.5)	0.6
Atopy,^†^ n (%)	41 (74.5)	31 (60.8)	0.1
Serum IgE (IU), median (IQR)	90 (204)	159 (487)	0.5
Former smoker, n (%)	14 (25.5)	14 (27.5)	0.8
Late-onset asthma,^‡^ n (%)	14 (25.5)	17 (33.3)	0.4
Duration of asthma symptoms (years), mean (95% CI)	37.1 (32.2-42.0)	34.6 (29.5-39.8)	0.5
Hospital admissions due to asthma, n (%)	21 (38.2)	40 (78.4)	< 0.001
ICU admissions due to asthma, n (%)	4 (7.3)	13 (25.5)	0.01
≥ 1 exacerbation in the last year, n (%)	19 (34.5)	36 (68.6)	< 0.001
BMI (kg/m^2^), mean, (95% CI)	27.1 (25.7-28.5)	30.2 (28.2-32.1)	0.01
Lung function parameters, mean (95% CI)			
Pre-BD FEV_1_, %	70.4 (65.5-75.3)	54.6 (48.4-60.7)	< 0.001
Post-BD FEV_1_, %	77.6 (73.1-82.2)	61.5 (55.3-67.7)	< 0.001
Pre-BD FVC, %	85.5 (81.4-89.5)	73.7 (68.4-79.0)	0.001
Post-BD FVC, %	91.2 (87.5-95.0)	81.0 (76.0-85.9)	0.001
Pre-BD FEV_1_/FVC	0.67 (0.64-0.70)	0.59 (0.55-0.62)	0.001
Post-BD FEV_1_/FVC	0.69 (0.66-0.73)	0.61 (0.57-0.64)	< 0.001
Δ Post-BD FEV_1_, %	12.1 (9.2-15.0)	17.8 (11.9-23.7)	0.001
Δ Post-BD FEV_1_, mL	190 (150-231.5)	195 (145.6-244.2)	0.5
Treatment			
ICS dose (µg/day), mean, (95% CI)	724 (681-766)	1,302 (1,196-1,408)	< 0.001
On LABA, n (%)	52 (94.5)	51 (100)	0,2
On LAMA, n (%)	1 (1.8)	9 (17.6)	0.007
On OCS, n (%)	0 (0)	5 (9.8)	0.02
On LTRA, n (%)	0 (0)	5 (9.8)	0.02
On biologics, n (%)	0 (0)	6 (11.8)	0.01
Courses of OCS in the last year (n), mean (95% CI)	0.7 (0.5-1.4)	2.9 (2.1-3.7)	< 0.001
Comorbidities			
Allergic rhinitis, n (%)	49 (89.1)	46 (90.2)	0.5
Gastroesophageal reflux, n (%)	30 (54.5)	26 (51.0)	0.6
Depression, n (%)	22 (40.0)	18 (35.3)	0.7
Hypertension, n (%)	20 (36.4)	17 (33.3)	0,8
Obesity, n (%)	16 (29.1)	21 (41.2)	0.1
Osteoporosis, n (%)	1 (1.8)	7 (13.7)	0.02
Cataracts, n (%)	3 (5.5)	9 (17.3)	0.05

BD: bronchodilator; ICS: inhaled corticosteroid; LABA: long-acting bronchodilator; LAMA: long-acting muscarinic antagonist; OCS: oral corticosteroid; and LTRA: leukotriene receptor antagonist. *Defined by the minimum amount of medication needed to maintain asthma control.^19^ ^†^One or more positive skin prick allergy tests, with a wheal > 2 mm larger than the negative control.^23^ ^‡^Onset of asthma symptoms at age >18 years.

In patients with SA, despite the use of high doses of ICS, OCS, or both, the key factors impacting health status included the number of previous hospital and ICU admissions and deterioration of lung function. Compared with the patients with SA, those with MA reported fewer hospital and ICU admissions (p < 0.001 for both). However, 12 (63.1%) of the 19 MA group patients who reported ≥ 1 exacerbation in the last year, corresponding to 21.8% of all of the patients with MA, had residual eosinophilia in IS at the time of the study visit, compared with 22 (62.8%) of the 35 such patients in the SA group, corresponding to 41.3% of all of the patients with SA. The number of exacerbations reported was not found to be associated with obesity, the type of inflammation, or the asthma phenotype.

**Figure 1:**
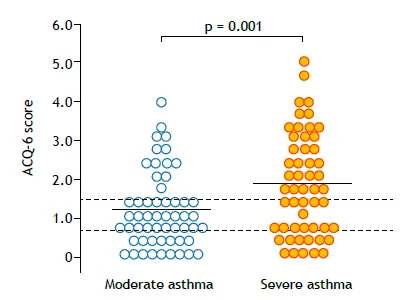
Asthma control, by disease severity. Horizontal bars represent the means of the six-item Asthma Control Questionnaire (ACQ-6) scores. The lower dotted line represents the cutoff for well-controlled asthma (< 0.75), and the upper dotted line represents the cutoff point for uncontrolled asthma (> 1.5). Note that, although there were patients with well-controlled asthma in both groups, there were significantly fewer patients with uncontrolled asthma in the moderate asthma group than in the severe asthma group (34.5% vs. 58.8%; p = 0.04).

Asthma control, as measured by the ACQ-6 score, demonstrated a significant, clinically important mean difference between the two groups (mean, 0.7; 95% CI: 0.3-1.2; p = 0.001), indicating worse outcomes in patients with SA, with differences between groups reaching the minimal clinically important difference (MCID) of 0.5 points (Figure 1). Patients with poorer asthma control at the time of the study visit were also significantly more likely to report ≥ 1 exacerbation in the last year than were those with controlled asthma at that time.

Regarding quality of life, as measured by the AQLQ(S), the differences between the MA and SA groups, in terms of the total AQLQ(S) score and the various AQLQ(S) domain scores, were statistically significant, except for the score on the environmental exposure domain. These differences were clinically relevant, as evidenced by the fact that they also exceeded the MCID (Table 2).

**Table 2:** Standardized Asthma Quality of Life Questionnaire results, by the level of asthma severity

AQLQ(S) result	Asthma intensity		Difference	p
	Moderate	Severe		
	(n = 55)	(n = 51)	Mean (95% CI)	
Total score, mean (95% CI)	5.1 (4.8-5.4)	4.5 (4.1-4.9)	0.6 (0.1-1.1)	0.02
Score by domain, mean (95% CI)				
Symptoms	5.3 (5.0-5.6)	4.7 (4.3-5.1)	0.6 (0.2-1.2)	0.01
Activity limitation	5.1 (4.8-5.5)	4.5 (4.1-4.,9)	0.6 (0.1-1.2)	0.02
Emotional function	5.0 (4.6-5.4)	4.3 (3.9-4.8)	0.7 (0.1-1.3)	0.04
Environmental stimuli	4.6 (4.2-5.1)	4.4 (3.9-5.0)	0.2 (0.4-0.9)	0.5

AQLQ(S): Standardized Asthma Quality of Life Questionnaire.

### IS cellularity versus bEOS

Table 3 shows the results for IS cellularity and bEOS in both groups. As expected, no differences were observed between the MA and SA groups. 

**Table 3:** Airway cellularity and blood eosinophils, by asthma severity.^a^

Characteristic	Asthma intensity		p
	Moderate	Severe	
	(n = 49)	(n = 46)	
Cell viability, %	69.4 (66.1-72.8)	73.5 (69.9-77.1)	0.1
Total cell count × 10^6^/mg	5.6 (4.4-6.7)	6.6 (4.8-8.4)	0.4
Neutrophils, %	57.4 (50.6-64.2)	59.7 (52.6-66.8)	0.5
Eosinophils, %	5.8 (1.7-9.9)	9.7 (4.4-15.1)	0.4
Macrophages, %	31.4 (25.4-37.3)	24.8 (19.8-29.7)	0.08
Lymphocytes, %	2.1 (1.8-2.5)	1.7 (1.3-2.0)	0.06
Blood eosinophils, cells/mm^3^	406 (298-514)	383.6 (306.6-460.6)	0.7

a Data expressed as mean (95% CI).

**Figure 2:**
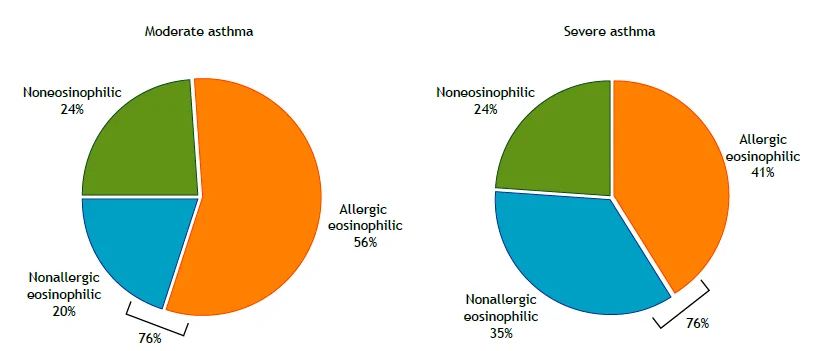
Asthma phenotypes in moderate and severe asthma. Although the most common phenotype was the eosinophilic phenotype in both groups, a significant proportion of the patients with moderate asthma (56%) had allergic eosinophilic asthma, compared with only 20% for nonallergic asthma (p < 0.001), whereas the prevalence of the two phenotypes was comparable between the patients with moderate asthma and those with severe asthma.

Figure 2 shows the different MA and SA phenotypes identified in the studied population. The eosinophilic phenotype was predominant, with a prevalence of 76% in both groups. However, the proportion of patients with the allergic eosinophilic phenotype was significantly greater in the MA group than in the SA group (56% vs. 20%; p < 0.001). Of the 106 patients in the study sample, only 25 (23.6%) had T2-low asthma. Of those 25 patients, 9 (36%) were classified as obese, 10 (40%) were former smokers, and only 3 (12%) had bronchiectasis.

**Figure 3:**
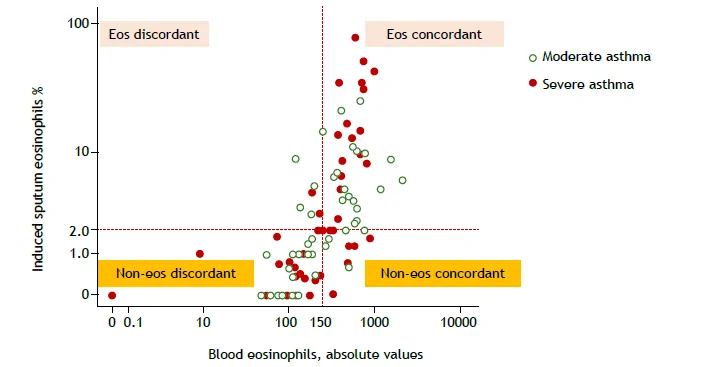
Induced sputum eosinophils and blood eosinophils in moderate and severe asthma. eos: eosinophilic. Open circles represent moderate asthma, and closed circles represent severe asthma. The horizontal dotted line represents the cutoff point for induced sputum eosinophils (> 2.0%), and the vertical dotted line represents the cutoff point for blood eosinophils (> 150 cells/mm^3^). In both groups (moderate and severe asthma), 76% of the patients had eosinophilia, as measured in induced sputum, peripheral blood, or both. The agreement between induced sputum eosinophils and blood eosinophils was moderate (κ = 0.519; p < 0.0001).

The association between IS eosinophils and bEOS was strong, with a correlation coefficient of 0.75, a coefficient of determination of 0.35 (p < 0.001), and a kappa statistic of 0.519 (p < 0.0001), indicating moderate agreement between IS eosinophils and bEOS. These findings are illustrated in Figure 3.

## DISCUSSION

In this study, we phenotyped subjects with MA and subjects with SA, matched by age and sex, analyzing IS cellularity, bEOS, and atopy. We found that the cellularity of IS and bEOS levels were comparable between the MA and SA groups. In both groups, the predominant phenotype was T2 (eosinophilic asthma, whether allergic or nonallergic, affecting 76% of the patients). Notably, the allergic eosinophilic phenotype was significantly more prevalent among the patients with MA. In addition, due to the inherent severity of their condition, individuals with SA reported a substantially greater burden of illness and a markedly poorer quality of life. Furthermore, over half of the subjects with MA or SA who reported experiencing one or more exacerbations in the past year exhibited sputum eosinophilia at the study visit. These results suggest the potential significance of timely biologic treatment to mitigate irreversible airway damage and remodeling, ultimately reducing the overall disease burden. However, this conclusion warrants validation through proper randomized controlled trials and requires further discussion.

Some findings of this study may not seem particularly surprising or novel. However, to our knowledge, this is the first comprehensive investigation comparing phenotypes of MA and SA within the context of Brazil. Previous studies in the literature have focused primarily on SA^32^ ^,^ ^33^ or have made comparisons between SA and non-severe asthma.^34^ In addition, in many randomized controlled trials involving patients with MA or SA, phenotyping results were often reported without differentiating subgroups based on severity. This distinction is critical because asthma severity exists on a continuum; patients at the upper end of MA may be at risk of progressing to SA. In this light, phenotyping MA earlier could be instrumental in identifying individuals at risk for worsening asthma due to factors predictive of frequent exacerbations (e.g., residual eosinophilic inflammation), accelerated loss of lung function, and airway remodeling. Key indicators may include eosinophilia in IS or peripheral blood, a history of exacerbations, decreased lung function, and poor asthma control.^35^

The largest asthma study conducted in Brazil investigated 385 patients with SA.^20^ The authors found that only 40% of the patients had an eosinophilic phenotype, as determined by measuring bEOS. Differences in bEOS cutoff points, measurements, and study populations may explain the discrepancies between our findings and those of that study. 

There is limited research on asthma phenotypes in adults that incorporates IS analysis and atopy assessment. A significant study conducted by de Carvalho-Pinto et al.^21^ characterized 74 patients with SA using IS analysis, fractional exhaled nitric oxide determination, atopy evaluation, pulmonary function testing (including plethysmography), and various control questionnaires, together with assessments of quality of life and depression.

Aligned with our findings, the de Carvalho-Pinto et al. study^21^ revealed that most patients with SA were women, were obese, had a long history of asthma symptoms/exacerbations, and had poor lung function. In their study, the IS analysis demonstrated that 79% of the patients with SA had eosinophilic asthma, and 64% were classified as atopic. However, the extent of overlap between eosinophilia and atopy in that cohort was not reported and there was no comparison with MA.

The distinction between atopic and nonatopic eosinophilic asthma is relevant in terms of the prognosis and response to treatment. Although both phenotypes were present in both groups of patients in our study, the proportion of patients with nonatopic eosinophilic asthma was higher in the SA group, suggesting that these patients have a worse prognosis. Although the observation that not all eosinophilic SA is related to atopy is not new, it challenges the traditional view of allergy as a key factor in the pathogenesis of SA, raising questions about alternative mechanisms that may contribute to eosinophilic inflammation in nonallergic SA.^36^

There is increasing evidence that type 2 innate lymphoid cells play a role in airway eosinophilia associated with nonallergic eosinophilic asthma.^37^ Patients with nonatopic SA typically experience late-onset asthma, recurrent exacerbations, and comorbidities such as sinusitis and nasal polyposis.^37^ Although that specific aspect was not directly investigated in our study, our findings align with those of de Carvalho-Pinto et al.,^21^ indicating that the proportion of patients with late-onset asthma is higher among those with SA.

We also observed that patients with MA did not differ significantly from those with SA regarding the most common comorbidities, except for obesity, osteoporosis, and cataracts, which were more prevalent in the SA group. Although osteoporosis and cataracts do not directly influence asthma control, they serve as indicators of the potential severity of treatment-related adverse effects. Notably, these conditions were found in younger patients, including one case that required early crystalline lens replacement. The side effects associated with high doses of ICS or OCS are among the primary reasons for initiating targeted biologic treatments at an earlier stage and must be considered, particularly in younger patients.

Another anticipated result of our study was the moderate agreement between IS cellularity and bEOS counts, given that the latter may be elevated because of factors other than eosinophilic airway inflammation.^8^ ^,^ ^24^ Nonetheless, a similarity between bEOS and IS eosinophils has been successfully used to indicate a positive response to treatment with biologics. Also, measuring bEOS is easier and simpler to perform than is induced sputum, as it does not require a specialized laboratory. These advantages have contributed to the widespread use of blood eosinophil counts/mm^3^ as a criterion for initiating biologic therapy in severe eosinophilic asthma.

In contrast, low eosinophil counts in peripheral blood can be influenced by various external factors, such as diurnal variation, recent infections, and different treatments. Therefore, it is recommended that at least three blood examinations on separate occasions be conducted to help rule out the eosinophilic phenotype.^8^ ^,^ ^24^

Our study has several strengths. First, it focused on a well-characterized population of patients with MA and SA. Another important strength was the use of IS analysis, bEOS counts, and atopy evaluation to phenotype the patients, which significantly increased the reliability of the asthma phenotype classification. All methods employed in this research have been validated, with IS analysis being considered the gold standard for assessing airway inflammation. Although the sample was relatively small in comparison with those of larger cohort studies, which could be considered a limitation, the sample size was calculated to adequately reject the null hypothesis of no differences between the inflammatory phenotypes of MA and SA. One potential limitation of our study is the cross-sectional design, that by its nature allowed us only to raise a hypothesis that requires further confirmation in randomized controlled trials. Nevertheless, this new hypothesis should not be ignored, despite requiring caution in its interpretation.

In conclusion, our analysis found that most subjects with SA or MA exhibited a T2 phenotype, predominantly of the allergic eosinophilic endotype in the MA group. We also observed significant residual eosinophilic airway inflammation and recurrent asthma exacerbations in approximately a quarter of the subjects with MA. These results highlight the need for further research on the optimal timing for initiating biologic treatments across different levels of asthma severity, particularly in patients with exacerbations and residual eosinophilia.
